# Supplementary material for: Identifying healthy individuals with Alzheimer’s disease neuroimaging phenotypes in the UK Biobank
Source: Commun Med (Lond). 2023 Jul 20;3:100. doi: 10.1038/s43856-023-00313-w (PMC10359360; doi:10.1038/s43856-023-00313-w)
Supplement: Supplementary file 2 — Supplementary Material [file 43856_2023_313_MOESM2_ESM.pdf]

SUPPLEMENTARY INFORMATION

Identifying healthy individuals with Alzheimer's disease neuroimaging phenotypes in the UK Biobank

Tiago Azevedo[1], Richard A.I. Bethlehem[2,3], David J. Whiteside[4], Nol Swaddiwudhipong[4], James B. Rowe[4], Pietro Lió[1], Timothy Rittman[4,*], and for the Alzheimer's Disease Neuroimaging Initiative[**]

[1]Department of Computer Science and Technology, University of Cambridge, Cambridge, UK
[2]Brain Mapping Unit, Department of Psychiatry, University of Cambridge, Cambridge, UK
[3]Autism Research Centre, Department of Psychiatry, University of Cambridge, Cambridge, UK
[4]Department of Clinical Neurosciences and Cambridge University Hospitals NHS Trust, University of Cambridge, Cambridge, UK
[*]tr332@medschl.cam.ac.uk
[**]Data used in preparation of this article were obtained from the Alzheimer's Disease Neuroimaging Initiative (ADNI) database (adni.loni.usc.edu). As such, the investigators within the ADNI contributed to the design and implementation of ADNI and/or provided data but did not participate in analysis or writing of this report. A complete listing of ADNI investigators can be found at: http://adni.loni.usc.edu/wp-content/uploads/how_to_apply/ADNI_Acknowledgement_List.pdf

Supplementary Notes 1

Uncertainty of AD score estimation in the neural network model

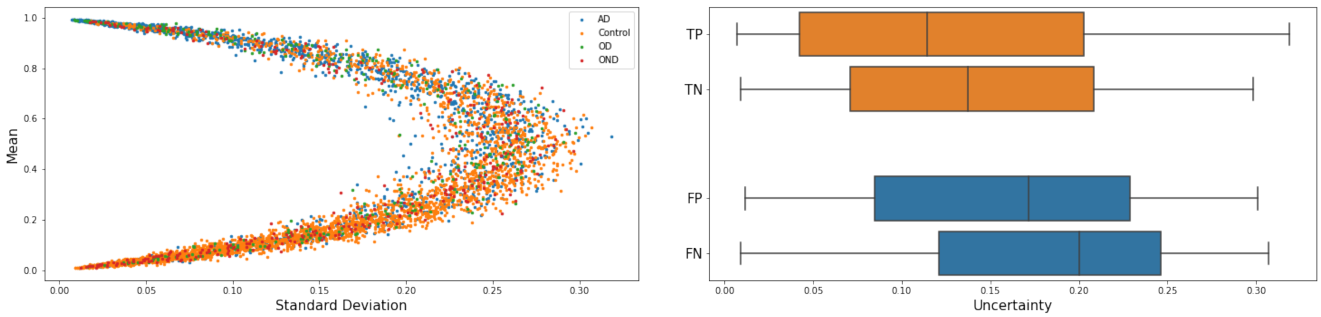

(a) (b)

Figure S1. Model uncertainty for the NACC dataset, where uncertainty was measured as the standard deviation of the model's sampled outputs. (a) Relation of model's output and uncertainty. The model was more certain (i.e. smaller standard deviation) for more extreme mean outputs. For a mean output closer to 0.5, more variable and generally greater uncertainty were seen. **(b)** Uncertainty levels for different categories in the confusion matrix applying a cut-off of 0.5, where TP=1168, TN=2574, FP=538, and FN=929. On average, uncertainty levels are higher for incorrect predictions (i.e. FP, FN) when compared to correct predictions (i.e. TP, TN). There was a significant difference among these four groups (Kruskal-Wallis H-test, $p < 3.79 \times 10^{-58}$).

Supplementary Notes 2

Comparison between Monte Carlo dropout and single forward pass

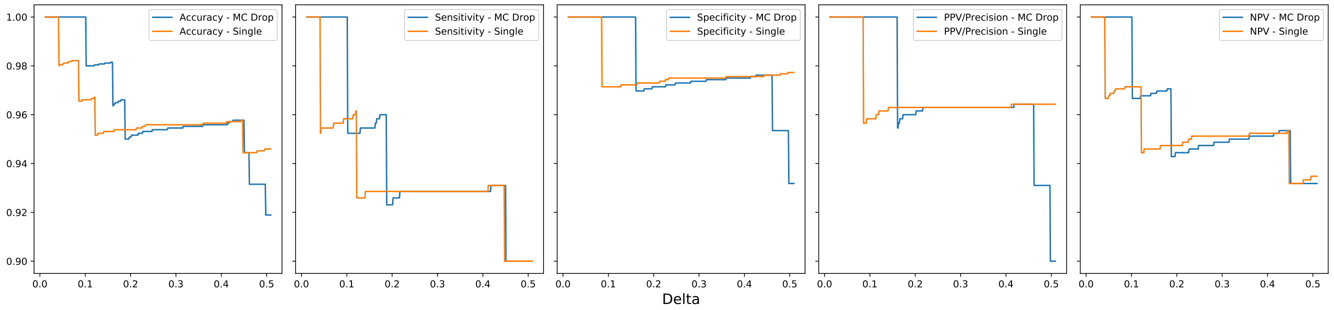

Figure S2. Comparison between our model with Monte Carlo dropout (with 50 samples), and a corresponding single forward pass one, for the ADNI test set. We sequentially evaluate performance by including different numbers of people according to the model's output, where delta corresponds to the distance to the model's extremes (ie. 0 or 1). As delta increases, more people are included with an output closer to 0.5. Metrics are calculated for a cut-off of 0.5.

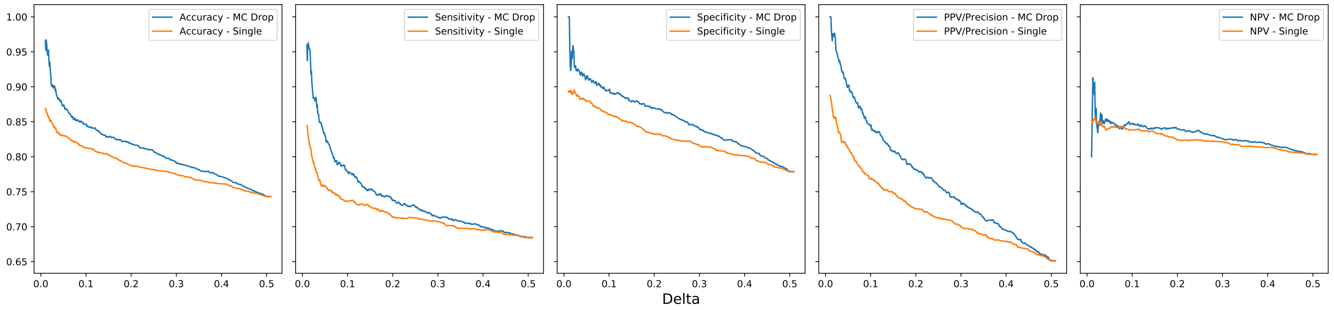

Figure S3. Comparison between our model with Monte Carlo dropout (with 50 samples), and a corresponding single forward pass one, for the NACC dataset with only AD and controls. We sequentially evaluate performance by including different numbers of people according to the model's output, where delta corresponds to the distance to the model's extremes (ie. 0 or 1). As delta increases, more people are included with an output closer to 0.5. Metrics are calculated for a cut-off of 0.5. Our model consistently outperforms the corresponding neural network with one forward pass.

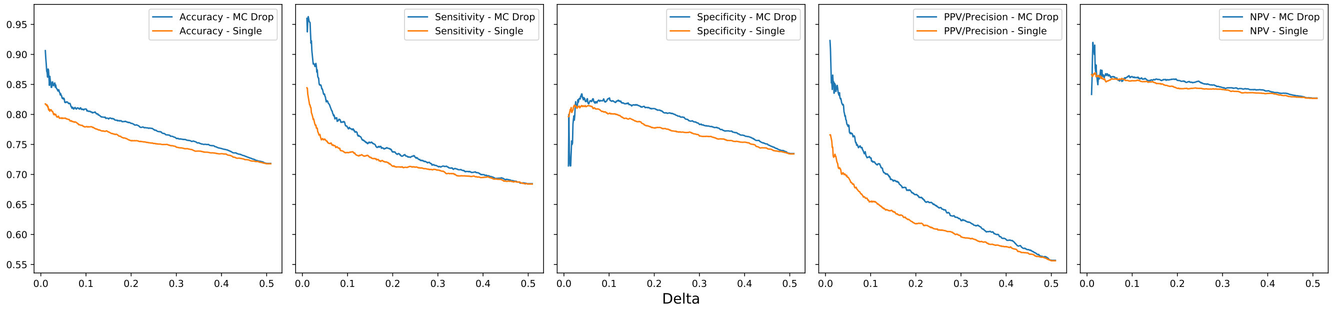

Figure S4. Comparison between our model with Monte Carlo dropout (with 50 samples), and a corresponding single forward pass one, for the NACC dataset including all diagnoses. We sequentially evaluate performance by including different numbers of people according to the model's output, where delta corresponds to the distance to the model's extremes (ie. 0 or 1). As delta increases, more people are included with an output closer to 0.5. Metrics are calculated for a cut-off of 0.5. Our model consistently outperforms the corresponding neural network with one forward pass.

Supplementary Notes 3

Model explainability

We investigated the potential explainability of our model using SHapley Additive exPlanations (SHAP)[1], a unified framework for interpreting predictions.

Figure S5 shows the aggregated feature impact on the model output in the ADNI validation set. In the figure, a point represents a sample from the dataset and its colour is the value of that feature rather than the importance on the model output. The y-axis contains the 20 most important input features, ranked by the aggregated magnitude of impact on the model output across all the samples (the 20th row is an aggregation of the contribution of all the remaining 136 features after the 19 most important). Each feature is assigned a SHAP value (in the x-axis) which represents the marginal impact (i.e., importance) on model output or, in other words, both the magnitude and direction of the feature's contribution. A higher SHAP value means that that feature contributed towards a higher predicted value in the model's output.

It is possible to interpret the contributions of individual brain regions to the model predictions. For instance, the cortical thickness of the left hemisphere's entorhinal area has an almost inverse effect in the model output: a lower value of this feature drives up Alzheimer's disease prediction with a similar magnitude as when a higher value of this feature drives the prediction down. This effect can be seen, as expected, in almost all the important features, with some exceptions (e.g., the cortical thicknesses of the right hemisphere's transverse temporal area, and the volume of the left hemisphere's precentral area).

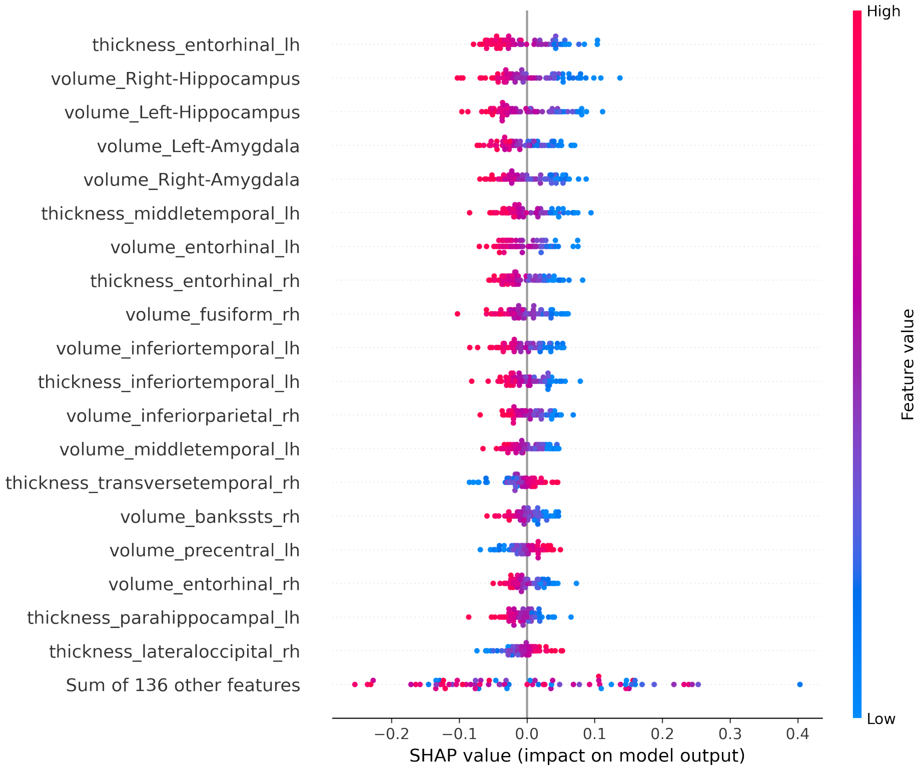

Figure S5. Contribution of the most important features across the ADNI validation set. For each feature represented in each row, vertical dispersion stands for the data points which share the same SHAP value for that feature. Each feature value is colour-coded from the highest (i.e. red) to the lowest value (i.e. blue). Higher SHAP values, which are distinct from the actual feature values, mean they contribute in a positive direction to the final predicted variable.

Besides allowing the interpretation and analysis of output drivers on an aggregated (i.e. global) level, SHAP also enables the analysis of individuals. As a reminder, SHAP values represent the change in the expected model prediction conditioned on each feature, therefore explaining the contribution of that feature towards the difference between the average model prediction and the actual final prediction.

In Figure S6 we show an example of the most important features driving the AD score in two individuals with a high AD score (0.957 in the top pane) and a low AD score (0.005 in the bottom pane). These plots decompose the drivers of predictions

[1] https://github.com/slundberg/shap

for one single sample each. The y-axis contains the most important features driving the prediction and the corresponding raw value in lighter grey, and the x-axis contains the SHAP value corresponding to the impact on final prediction from the baseline prediction across the population (represented by $E[f(X)]$). The SHAP value of each individual feature is detailed in the arrows that move the prediction from the $E[f(X)]$ baseline. A striking difference between the two plots is that for the top one, the most important features drive most of the output value, but in the sample on the bottom, the remaining 146 other features (in total) have a much greater effect. This could point an expert to a more wider analysis on the whole brain (in the bottom case), while the analysis on the top case can possibly be more focused on a handful of brain regions.

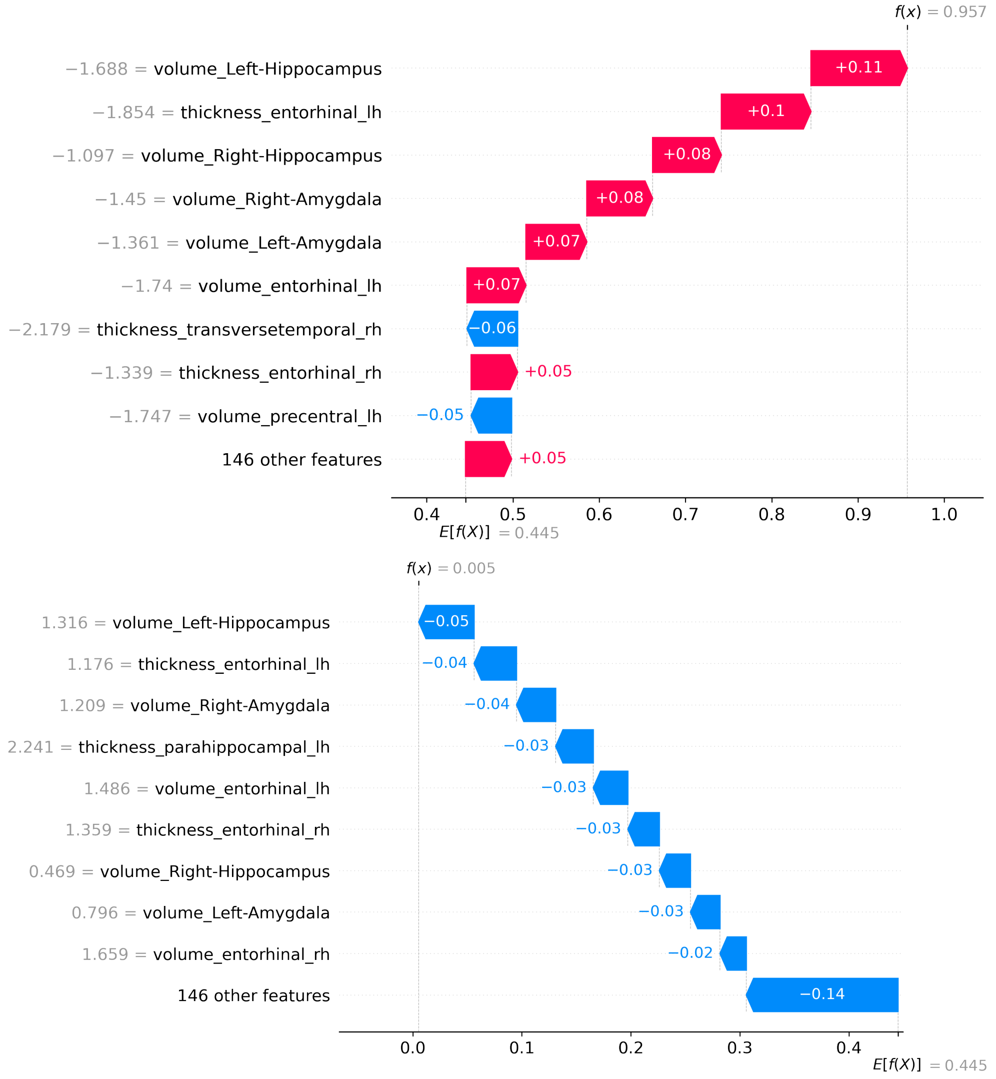

Figure S6. Contribution of the most important features in two samples of the ADNI validation set. The most important features driving different final outputs in two distinct people with a high (above) and low (below) AD score.

Training on a balanced ADNI training set

We employed the same training pipeline (i.e., same preprocessing steps and hyperparameters) with a balanced training ADNI set (i.e., by removing 60 control people to have 301 people both in the AD and control groups), and evaluated this new model on the ADNI and NACC validation sets. Resulting metrics can be seen on table S1. Overall, metrics are very similar or slightly worse when compared to the model trained on the unbalanced dataset (with the exception of the sensitivity metric), which is expected as we are reducing the number of training samples on an already small dataset.

Table S1. Performance metrics across datasets with a model trained on a balanced ADNI training set (i.e., same number of people with AD diagnosis and controls), using a cut-off of and AD score of 0.5 and employing inference using MC Dropout with 50 samples. AUC=Area under the ROC curve. PPV=Positive predictive value. NPV=Negative predictive value. Results with the unbalanced (i.e., original) dataset presented for comparison.

Dataset	Accuracy	AUC	Sensitivity	Specificity	PPV/Precision	NPV
ADNI test set [unbalanced]	0.92	0.97	0.90	0.93	0.90	0.93
ADNI test set [balanced]	0.89	0.97	0.87	0.91	0.87	0.91
NACC (only AD/Control) [unbalanced]	0.74	0.79	0.68	0.78	0.65	0.80
NACC (only AD/Control) [balanced]	0.74	0.79	0.7	0.76	0.64	0.81
NACC (AD/All) [unbalanced]	0.72	0.76	0.68	0.73	0.56	0.83
NACC (AD/All) [balanced]	0.71	0.76	0.7	0.72	0.55	0.83

Supplementary Notes 4

Predictive power of hippocampal volume

We fitted a linear regression model to the training (ADNI) set using ordinary least squares (OLS), in which the dependent variable was AD diagnosis, and independent variables were left hippocampus volume, right hippocampus volume, age, estimated total intracranial volume, and sex. This model was then employed on the ADNI test set, and resulting metrics can be seen in table S2.

Table S2. Performance metrics evaluated on ADNI test set using the main model presented in the paper, and a linear regression model based on hippocampal volume. AUC=Area under the ROC curve. PPV=Positive predictive value. NPV=Negative predictive value.

Model	Accuracy	AUC	Sensitivity	Specificity	PPV/Precision	NPV
Deep Learning	0.92	0.97	0.90	0.93	0.90	0.93
Linear regression	0.80	0.80	0.77	0.82	0.74	0.84

Supplementary Notes 5

NACC clinical scores

Table S3. The association between NACC MMSE scores and AD scores with a variable breakpoint.

	mean	se_mean	sd	2.5%	25%	50%	75%	97.5%	n_eff	Rhat
intercept	0.081	0.0027	0.097	-0.11	0.016	0.08	0.15	0.27	1332	1.00
bp	0.67	0.0021	0.084	0.43	0.64	0.7	0.73	0.75	1689	1.00
slope_before	-0.19	0.0017	0.069	-0.33	-0.24	-0.19	-0.15	-0.054	1628	1.00
slope_after	-1.4	0.0053	0.22	-1.8	-1.5	-1.3	-1.2	-0.94	1705	1.00
slope_difference	-1.2	0.0066	0.26	-1.7	-1.3	-1.2	-0.98	-0.65	1585	1.00
error	0.87	0.00058	0.028	0.82	0.85	0.87	0.88	0.92	2247	1.00

Table S4. The association between NACC MMSE scores and AD score with a breakpoint of the AD score fixed at 0.5

	mean	se_mean	sd	2.5%	25%	50%	75%	97.5%	n_eff	Rhat
intercept	0.15	0.0026	0.096	-0.036	0.083	0.14	0.21	0.34	1316	1.00
slope_before	-0.17	0.0021	0.077	-0.32	-0.23	-0.17	-0.12	-0.022	1350	1.00
slope_after	-1.1	0.004	0.15	-1.4	-1.2	-1.1	-1	-0.82	1418	1.00
slope_difference	-0.95	0.006	0.21	-1.4	-1.1	-0.94	-0.81	-0.53	1274	1.00
error	0.87	0.00059	0.026	0.82	0.85	0.87	0.89	0.92	1970	1.00

Table S5. A linear model of NACC MMSE scores and AD scores with no breakpoint.

	mean	se_mean	sd	2.5%	25%	50%	75%	97.5%	n_eff	Rhat
intercept	0.081	0.0027	0.097	-0.11	0.016	0.08	0.15	0.27	1332	1.00
bp	0.67	0.0021	0.084	0.43	0.64	0.7	0.73	0.75	1689	1.00
slope_before	-0.19	0.0017	0.069	-0.33	-0.24	-0.19	-0.15	-0.054	1628	1.00
slope_after	-1.4	0.0053	0.22	-1.8	-1.5	-1.3	-1.2	-0.94	1705	1.00
slope_difference	-1.2	0.0066	0.26	-1.7	-1.3	-1.2	-0.98	-0.65	1585	1.00
error	0.87	0.00058	0.028	0.82	0.85	0.87	0.88	0.92	2247	1.00

Table S6. The association between NACC MoCA scores and AD scores with a variable breakpoint.

	mean	se_mean	sd	2.5%	25%	50%	75%	97.5%	n_eff	Rhat
intercept	0.081	0.0027	0.097	-0.11	0.016	0.08	0.15	0.27	1332	1.00
bp	0.67	0.0021	0.084	0.43	0.64	0.7	0.73	0.75	1689	1.00
slope_before	-0.19	0.0017	0.069	-0.33	-0.24	-0.19	-0.15	-0.054	1628	1.00
slope_after	-1.4	0.0053	0.22	-1.8	-1.5	-1.3	-1.2	-0.94	1705	1.00
slope_difference	-1.2	0.0066	0.26	-1.7	-1.3	-1.2	-0.98	-0.65	1585	1.00
error	0.87	0.00058	0.028	0.82	0.85	0.87	0.88	0.92	2247	1.00

Table S7. The association between NACC MMSE scores and AD score with a breakpoint of the AD score fixed at 0.5

	mean	se_mean	sd	2.5%	25%	50%	75%	97.5%	n_eff	Rhat
intercept	0.15	0.0026	0.096	-0.036	0.083	0.14	0.21	0.34	1316	1.00
slope_before	-0.17	0.0021	0.077	-0.32	-0.23	-0.17	-0.12	-0.022	1350	1.00
slope_after	-1.1	0.004	0.15	-1.4	-1.2	-1.1	-1	-0.82	1418	1.00
slope_difference	-0.95	0.006	0.21	-1.4	-1.1	-0.94	-0.81	-0.53	1274	1.00
error	0.87	0.00059	0.026	0.82	0.85	0.87	0.89	0.92	1970	1.00

Supplementary Notes 6

Validation of the an AD cut-off score of 0.5

We investigated the association of AD score with clinical scores using piecewise linar regression models both with an without a breakpoint, and a linear regression model with no breakpoint. In the flexible breakpoint model, the breakpoint was restricted to between 0.25 and 0.75 to avoid improbable extreme values. We report the comparisons in model fit between the models. For each comparison one of the models 'wins', indicated in bold in table S8 with a model fit (Expected Log Probability Density Function, ELPDF) score of 0. The difference in model fit is expressed as a negative ELPDF. A model can be considered substantially worse if the magnitude of the difference is large and the standard deviation of the ELPDF is substantially smaller than the difference in model fit. This pattern is seen in MMSE, MoCA and semantic fluency for the linear regression model without a breakpoint, demonstrating that the two piecewise regression models are broadly equivalent, but both piecewise regression models are a substantially better fit than the simple linear regression model without a breakpoint.

There was a slightly better model fit in all cases for a variable rather than fixed breakpoint, however there was only weak evidence for this given that the standard deviation for the expected log probability density function was approximately similar to the difference in model fit. Reassuringly, there was no convincing superiority of the piecewise regression models over linear regression models in forward and backward digit span (that did not show differences between AD positive and negative scores), suggesting that the piecewise regression models did not overfit the data.

Table S8. ELPDF = Expected Log Probability Density Function, sd = Standard deviation.

	Fixed breakpoint (0.5)	Variable breakpoint		Linear model
	ELPDF (sd)	Breakpoint	ELPDF (sd)	ELPDF (sd)
MMSE	-1.3 (1.0)	0.67	**0.0 (0)**	-10.0 (5.3)
MoCA	-0.5 (0.6)	0.60	**0.0 (0)**	-12.4 (6.8)
Digit span (forward)	-0.4 (0.3)	0.58	**0.0 (0)**	-2.6 (2.6)
Digit span (backward)	-0.2 (0.2)	0.56	**0.0 (0)**	-0.5 (1.6)
Semantic fluency	-0.5 (0.4)	0.59	**0.0 (0)**	-9.6 (4.5)
Trails B	**0.0 (0.2)**	0.44	**0.0 (0)**	-2.7 (2.8)
WAIS	**0.0 (0)**	0.53	-0.1 (0.1)	-0.3 (1.4)
Boston naming task	-1.7 (0.7)	0.66	**0.0 (0)**	-9.6 (4.6)
