# Supplementary material for: Identifying healthy individuals with Alzheimer’s disease neuroimaging phenotypes in the UK Biobank
Source: Commun Med (Lond). 2023 Jul 20;3:100. doi: 10.1038/s43856-023-00313-w (PMC10359360; doi:10.1038/s43856-023-00313-w)
Supplement: Supplementary file 3 — Reporting Summary [file 43856_2023_313_MOESM3_ESM.pdf]

nature portfolio

Corresponding author(s): DBPR COMMSMED-21-0614

Last updated by author(s): Mar 15, 2023

# Reporting Summary

Nature Portfolio wishes to improve the reproducibility of the work that we publish. This form provides structure for consistency and transparency in reporting. For further information on Nature Portfolio policies, see our Editorial Policies and the Editorial Policy Checklist.

## Statistics

For all statistical analyses, confirm that the following items are present in the figure legend, table legend, main text, or Methods section.

| n/a | Confirmed | |
|---|---|---|
| ☐ | ☒ | The exact sample size (*n*) for each experimental group/condition, given as a discrete number and unit of measurement |
| ☐ | ☒ | A statement on whether measurements were taken from distinct samples or whether the same sample was measured repeatedly |
| ☐ | ☒ | The statistical test(s) used AND whether they are one- or two-sided<br>*Only common tests should be described solely by name; describe more complex techniques in the Methods section.* |
| ☐ | ☒ | A description of all covariates tested |
| ☐ | ☒ | A description of any assumptions or corrections, such as tests of normality and adjustment for multiple comparisons |
| ☐ | ☒ | A full description of the statistical parameters including central tendency (e.g. means) or other basic estimates (e.g. regression coefficient) AND variation (e.g. standard deviation) or associated estimates of uncertainty (e.g. confidence intervals) |
| ☒ | ☐ | For null hypothesis testing, the test statistic (e.g. *F*, *t*, *r*) with confidence intervals, effect sizes, degrees of freedom and *P* value noted<br>*Give P values as exact values whenever suitable.* |
| ☐ | ☒ | For Bayesian analysis, information on the choice of priors and Markov chain Monte Carlo settings |
| ☒ | ☐ | For hierarchical and complex designs, identification of the appropriate level for tests and full reporting of outcomes |
| ☐ | ☒ | Estimates of effect sizes (e.g. Cohen's *d*, Pearson's *r*), indicating how they were calculated |

*Our web collection on statistics for biologists contains articles on many of the points above.*

## Software and code

Policy information about availability of computer code

| | |
|---|---|
| Data collection | ADNI, NACC, UK biobank |
| Data analysis | All code is available on github as outlined in the paper. |

For manuscripts utilizing custom algorithms or software that are central to the research but not yet described in published literature, software must be made available to editors and reviewers. We strongly encourage code deposition in a community repository (e.g. GitHub). See the Nature Portfolio guidelines for submitting code & software for further information.

## Data

Policy information about availability of data

All manuscripts must include a data availability statement. This statement should provide the following information, where applicable:

- Accession codes, unique identifiers, or web links for publicly available datasets
- A description of any restrictions on data availability
- For clinical datasets or third party data, please ensure that the statement adheres to our policy

All the data used in this study is available by application to the data managers of ADNI, NACC or the UK Biobank. ADNI data used in the preparation of this article were obtained from the Alzheimer's Disease Neuroimaging Initiative (ADNI) database (adni.loni.usc.edu). The ADNI was launched in 2003 as a public-private partnership, led by Principal Investigator Michael W. Weiner, MD. The primary goal of ADNI has been to test whether serial magnetic resonance imaging (MRI), positron emission tomography (PET), other biological markers, and clinical and neuropsychological assessment can be combined to measure the progression of mild

cognitive impairment (MCI) and early Alzheimer's disease (AD). For up-to-date information, see www.adni-info.org.The data generated by this work cannot be directly shared due to restrictions set by each consortium.

Code (for reproducibility) and results are publicly available on Github, with additional instructions for implementation: https://github.com/tjiagoM/adni_phenotypes (https://doi.org/10.5281/zenodo.7738962)

## Human research participants

Policy information about studies involving human research participants and Sex and Gender in Research.

| | |
|---|---|
| Reporting on sex and gender | Sex is reported for the datasets |
| Population characteristics | No population characteristics required adjusting for in this project. |
| Recruitment | Participants were recruited as outlined in the ADNI, NACC or UK biobank projects. |
| Ethics oversight | Ethical approval was arranged for each of the ADNI, NACC and UK biobank datasets separately. No additional ethica approval was required for this analysis. |

Note that full information on the approval of the study protocol must also be provided in the manuscript.

# Field-specific reporting

Please select the one below that is the best fit for your research. If you are not sure, read the appropriate sections before making your selection.

☒ Life sciences ☐ Behavioural & social sciences ☐ Ecological, evolutionary & environmental sciences

For a reference copy of the document with all sections, see nature.com/documents/nr-reporting-summary-flat.pdf

# Life sciences study design

All studies must disclose on these points even when the disclosure is negative.

| | |
|---|---|
| Sample size | Sample size was determined by the availability of data. Calculating sample size for machine learning algorithms is cahllenging, however we wough to include all available data. |
| Data exclusions | Participants in the UK biobank population were excluded if they already had a diagnosis of dementia. |
| Replication | The findings in the ADNI dataset were independently validated in the NACC dataset. |
| Randomization | N/A |
| Blinding | N/A |

# Reporting for specific materials, systems and methods

We require information from authors about some types of materials, experimental systems and methods used in many studies. Here, indicate whether each material, system or method listed is relevant to your study. If you are not sure if a list item applies to your research, read the appropriate section before selecting a response.

## Materials & experimental systems

| n/a | Involved in the study | |
|---|---|---|
| ☒ | ☐ | Antibodies |
| ☒ | ☐ | Eukaryotic cell lines |
| ☒ | ☐ | Palaeontology and archaeology |
| ☒ | ☐ | Animals and other organisms |
| ☒ | ☐ | Clinical data |
| ☒ | ☐ | Dual use research of concern |

## Methods

| n/a | Involved in the study | |
|---|---|---|
| ☒ | ☐ | ChIP-seq |
| ☒ | ☐ | Flow cytometry |
| ☐ | ☒ | MRI-based neuroimaging |

## Magnetic resonance imaging

### Experimental design

| | |
|---|---|
| Design type | Structural MRI freesurfer (volume/thickness) analysis. |
| Design specifications | N/A |
| Behavioral performance measures | N/A |

### Acquisition

| | |
|---|---|
| Imaging type(s) | T1/MPRAGE structural MRI |
| Field strength | 1.5T - 3T |
| Sequence & imaging parameters | Variable - see individual study protocols. |
| Area of acquisition | Whole brain |

Diffusion MRI ☐ Used ☒ Not used

### Preprocessing

| | |
|---|---|
| Preprocessing software | Freesurfer version 6 and version 7 |
| Normalization | N/A |
| Normalization template | N/A |
| Noise and artifact removal | N/A |
| Volume censoring | N/A |

### Statistical modeling & inference

| | |
|---|---|
| Model type and settings | Deep learning classification model. |
| Effect(s) tested | N/A |

Specify type of analysis: ☐ Whole brain ☐ ROI-based ☒ Both

Anatomical location(s): *Describe how anatomical locations were determined (e.g. specify whether automated labeling algorithms or probabilistic atlases were used).*

| | |
|---|---|
| Statistic type for inference (See Eklund et al. 2016) | N/A |
| Correction | N/A |

### Models & analysis

| n/a | Involved in the study |
|---|---|
| ☒ | ☐ Functional and/or effective connectivity |
| ☒ | ☐ Graph analysis |
| ☒ | ☐ Multivariate modeling or predictive analysis |
